# Current opinions concerning the restoration of endodontically treated teeth: basic principles

**Published:** 2009-04-25

**Authors:** C VȦrlan, B Dimitriu, V VȦrlan, D Bodnar, I Suciu

**Affiliations:** * ‘Carol Davila’ University of Medicine and Pharmacy Bucharest, Faculty of Dental Medicine, Department of Operative DentistryRomania; ** ‘Carol Davila’ University of Medicine and Pharmacy Bucharest, Faculty of Dental Medicine, Department of Endodontics Romania

**Keywords:** Endodontically treated teeth, criteria for coronal restoration, functional occlusal forces, reconstruction materials and techniques

## Abstract

The goal of this general article is to present a survey of the current knowledge about the clinical approach of restoring endodontically treated teeth.

The best way to restore teeth after root canal treatment has long been and still is a controversial subject of debate to this day.

The clinical approach of restoring endodontically treated teeth needs taking into consideration several issues: aims of coronal restoration, criteria for establishing the various modalities of coronal restoration,  clinical solutions of restoring teeth after endodontic treatment, guidelines regarding restorative materials and techniques, possibilities and limits of restoration using direct adhesive materials and techniques.

The aims of coronal restoration of endodontically treated teeth are generally considered to be the following ones: to prevent recontamination of the root canal system and / or periapical space, to replace missing hard dental tissues and to restore coronal morphology and functions, to provide the necessary strength for the restoration/tooth complex in order to withstand functional stress and prevent crown and/or root fracture.

The criteria for establishing the modalities of coronal restoration for endodontically treated teeth are: amount and quality of remaining hard dental tissues, topography and coronal morphology of the tooth,  functional occlusal forces that the restoration/tooth complex has to withstand, restoring requirements in order to include the treated tooth in a comprehensive oral rehabilitation treatment plan, esthetic requirements.

## Introduction

A lot of different parameters which influence the prognosis  of endodontically treated teeth have to be taken into consideration: apical status, position of the tooth in the dental arch, number of adjacent teeth, occlusal contacts, amount of hard tissue loss, remaining dentin wall thickness, collagen degradation and intermolecular cross linking of the root dentin, type of long–term coronal restoration, type of post (only if needed) and core material used, presence, if necessary, of a ferrule preparation [1].

Coronal restoration of endodontically treated teeth may be considered one of the main aforementioned parameters, since it represents a major concern, for both practitioner and patient.

The best way to restore teeth after root canal treatment has long been and still is a controversial subject of debate to this day. To begin with the end in mind, it seems to be the most appropriate plan for success [2]. Before initiating endodontic treatment, the tooth should be assessed for restorability, occlusal function, and periodontal health, and aspects such as biological width and crown–to– root ratio should be evaluated. If satisfactory, these factors will allow the tooth to be included in a comprehensive oral rehabilitation treatment plan [3].

The advisable clinical approach is to completely remove previous restorations and all existing caries before initiating root canal treatment, therefore a more accurate evaluation of the tooth status will be possible. Extensive absence of sound hard dental tissues leading to important coronal destruction often requires surgical crown lengthening or orthodontic eruption prior to endodontic treatment, in order to fulfill the basic principles of endodontically treated teeth restoration. Thus, the adequate guidelines for the root canal treatment will be upheld [3].

*‘Contamination of the root–canal system by saliva, often referred to as ‘coronal leakage’ or ‘coronal microleakage’, is a potential cause of endodontic failure[4 cit. by 5].’* The aforecited assertion shows that an important cause of future problems for endodontically treated teeth is considered to be the contamination of the root canal system between completion of endodontic treatment and restoration of the tooth. In order to prevent such problems, a main concern should be to immediately restore the tooth. Sometimes the procedures for a long–term restoration are delayed, because of the time considered to be necessary for the assessment of the endodontic treatment success. This is not the best approach, since temporary restorations do not effectively prevent contamination for extended periods of time.

When immediate restoration is not possible, the root canal system should be protected from saliva contamination. Orifice sealing using bonded materials such as composite resin or glass ionomer cements are usually recommended choices. Traditional temporary materials, such as IRM, Cavit, Citodur, Fermin, used for the coronal access cavity, do not protect the tooth against fracture and the practitioner has to be aware that such temporary restoration should be avoided for prolonged time.

This article aims to provide a review of the basic principles for restoring endodontically treated teeth, as mentioned in literature, and to highlight the most significant aspects of clinical procedures, upon which restoration guidelines are based.

## Significance of remaining coronal tooth structure

The amount of remaining tooth structure is probably the single most important predictor of clinical success [6]. I n most cases, it is limited as a result of trauma, caries, prior restoration and endodontic procedures, reducing the fracture resistance of the tooth. Endodontic access in combination with the earlier loss of one or both marginal ridges leave the tooth at serious risk of fracture, even if it was reduced out of direct occlusal contact before endodontic treatment began. The post design probably has a limited role in the fracture resistance of the restored tooth, if more than 2mm of tooth structure remains [7].

Furthermore, the strength of an endodontically treated tooth is reported to be directly related to the bulk of remaining dentine.

To ensure functional longevity, endodontically treated teeth must have at least 5 mm of tooth structure coronal to the crestal bone: 3mm are needed to maintain a healthy soft tissue complex and 2mm of coronal tooth structure incisal to the preparation finish line are necessary to ensure structural integrity.

When remaining coronal tooth structure is less than 5mm in height, it may be increased either surgically through a crown lengthening procedure or orthodontically through forced extrusion of the tooth. Both procedures result in a satisfactory and predictable increase in coronal tooth structure but may not be recommended in situations in which the crown–to–root ratio is compromised or where further exposure of tooth structure will have adverse esthetic results. As coronal tooth structure is increased by crown lengthening, the corresponding osseous-supported tooth structure is decreased. This change in the crown–to–root ratio may render the tooth less resistant to lateral forces. A 1:1 crown–to– root ratio has been advocated as the minimum ratio necessary for resisting lateral forces that may occur during function [8].

There is convincing evidence that cuspal coverage after root canal treatment should be provided for posterior teeth. Access preparations result in greater cuspal flexure, increasing the probability of cuspal fracture. The presence of cuspal coverage is the only significant restorative variable to predict long–term success for such teeth. This conclusion is based on an independent, retrospective study of 608 endodontically treated teeth that evaluated the factors that affected survival during a 10–years period [9]. Another retrospective study of 400 teeth during a 9–years period found that endodontically treated teeth with cuspal coverage were six times more likely to survive than those with intracoronal restorations [10]. A further argument for cuspal coverage comes from a survey in private dental offices, reporting that ‘unfavorable’ subgingival fractures occurred more often in endodontically treated teeth [11].

On the other hand, a study regarding endodontically treated teeth restored with fiber posts and composite showed no difference in failures, with or without cuspal coverage. Nevertheless, the survey time was only 3 years, which may not be long enough to detect differences in failure rates [ 12].

Despite evidence of the benefits of cuspal coverage, only about 50% of endodontically treated posterior teeth were found to be restored with cuspal coverage restorations [5].

When direct bonded restorations are not suitable, a core build–up followed by a partial or a complete crown coverage will be considered and the presence of a ferrule is needed. The cervical zone of a complete crown restoration functions like a ferrule when interfacing with 360^Ŷ^ of complete circumferential tooth structure between the core and preparation finish line. Endodontically treated teeth often have insufficient coronal tooth structure due to extensive destruction of the tooth by the carious process. The ferrule effect is a feature of the crown restoration encircling tooth structure [13]. This ferrule effect has been shown to provide positive reinforcement to endodontically treated teeth by resisting leveraged functional forces, the wedging effect of tapered posts, and lateral forces exerted during post insertion. It has been demonstrated that 1.5 mm of axial wall height significantly enhances endodontically treated teeth restored with cast posts and cores and complete crowns. For endodontically treated teeth restored with prefabricated posts, composite resin cores, and complete crowns, it has been reported that 2.0 mm of axial wall height beneficially increased their fracture resistance [7].

It has also been  demonstrated that the presence of remaining coronal tooth structure between the core and preparation finish line was more important for fracture resistance of endodontically treated teeth than post length or type [8].

## Significance of occlusal forces

One of the main goals of endodontic treatment is to ensure a clinical symptom free functional condition for the tooth. The need for a proper occlusal equilibration during and following endodontic and restorative treatment is mandatory.

Occlusal forces have been intensively investigated for a long time, but the approach and accuracy of measurement have been constantly improved over time. The initial finding: ‘The functional chewing forces are small compared to static isometric closing forces that the stomatognathic system can exert’ still stands. The first report about masticatory force was published in 1956 [14] and showed that normal force varied by the consistency of the food being chewed between 71–142 N. More recent research proved that the magnitude of masticatory forces ranges from 9 to 180 N, with a duration of 0.25–0.33 seconds. Maximum biting force in young subjects has been found to be 516–532 N. Bite force was not affected by the presence of restorations, but was influenced by gender: 847 N for men versus 597 N for women (mean maximum bite force). The maximum bite force in patients who bruxed was 911 N in the molar region of men versus 569 N in the incisor region. Whatever the actual values, it is apparent that the most extreme forces are in the most posterior teeth. When calculated as force per area and then converted to international units, a force of 911 N affecting a point of contact of 0,201cm^2^ places 45.23 MPa of force. Normal chewing force using the same area of contact results in a force of 8.826 KPa, well below the modulus of elasticity of dentin and the one of most contemporary direct adhesive restorative materials [15].

Endodontically treated teeth can withstand a maximum bite force comparable to natural teeth, being therefore able to regain a level of masticatory function similar to that in sound teeth [16].

The maximum bite force goes down if posterior teeth are lost and the proprioception is altered. Nocturnal bite force of bruxing is different from daytime voluntary maximum bite force:  220N (mean) and 415N (maximum), versus 775 N. Measured nocturnal bruxing forces last 7.1 seconds versus the normal chewing duration of 0.25–0.33 seconds. The longer duration of bruxing with greater force than used for chewing could cause greater damage to the teeth restored after root canal treatment.

Clenching force on one tooth is reported to be up to ten times greater than maximum biting forces distributed in a balanced way. Maximum biting forces are exerted in the maximum intercuspal position and are distributed according to distance from the condyles: the second molar takes 55% of the maximum force, while the incisors take only 20%. Research demonstrates that, due to progressive cuspal displacement both time– and load–dependent, continuous loading as in clenching is more destructive than cyclic loading as in chewing [15].

Normal chewing, single– and/or multiple–tooth bruxing and clenching exert variable effects on the restored endodontically treated teeth. Photoelastic studies showed that distal slopes of cusps and lingual slopes of the buccal cusps received the greatest force on mandibular molars. A flat plane occlusion considerably increases the stress on the teeth. In order to decrease the magnitude of the stress, it is advisable to maintain occlusal points of contact with opposing teeth instead of areas of occlusal contact. Non–axial forces create a greater risk for fatigue fractures of pulp less teeth, especially those reconstructed with dowel and cores [17]. A favorable occlusal design is more important for the longevity of restored pulp less teeth than is the type of post used.

It is well known that occlusal forces can bend teeth to some degree. This deformation is normally elastic. However, continuous loading, especially in restored teeth, can cause permanent deformation, leaving dentinal cracks and tears. With continued use and aging, these dentinal cracks can propagate causing the fracture of a part of the tooth.

As a mechanism to protect teeth from fracture, dental pulp contains mechano–receptors that are used to subconsciously limit the maximum biting force and consciously detect hardness differences during chewing. Moreover, the periodontal ligament mechano–receptors can encode the intensity of both steady forces and the rate of the force as it increases. On the other hand, intradental mechano-receptors, located in the tooth root, provide the same sensations as the periodontal ligament mechano-receptors [15].

The displacement of the periodontal ligament caused by occlusal stress due to normal and paranormal function (mastication, clenching, bruxing) can vary, allowing the teeth to move. Endodontically treated teeth retain the natural periodontal ligament, which allows physiologic movement. These teeth can respond and adapt to functional occlusal forces to permit maximum occlusal contact during biting [18].

These mechanisms have to be taken into account in specific clinical situations when selecting materials and techniques for single–tooth coronal restoration or abutment teeth reconstruction following root canal treatment,  scientifically based to ensure tooth longevity [1^19^].

## Significance of coronal microleakage

Coronal micro leakage is considered a major cause of endodontic failure, besides the traditional causes, which include poor apical seal and poor canal debridation and obturation. Saliva and microorganisms from the oral cavity may rapidly migrate alongside poorly adapted coronal restorations and even root fillings. The periradicular tissues will become inflamed by such reinfection and microorganisms lying dormant after initial treatment may be reactivated. A well–sealed coronal restoration is therefore critical to endodontic success, and it is emphasized that this applies as strongly to temporary restorations as it does to permanent ones. In addition, recurrent caries or fractured restorations may lead to recontamination of the root canal system [3].

Under the best of conditions, the oral environment is rich in microorganisms, and dental restorations must withstand repeated exposure to physical, chemical, and thermal stress factors. It is a difficult environment in which to maintain a hermetically sealed system. Exposure of coronal gutta–percha to bacterial contamination can lead to migration of bacteria to the apex in a matter of days. Bacterial byproducts and endotoxins can penetrate to the apex in an even shorter time than bacteria. Retreatment should be considered when the root canal space has been grossly and persistently contaminated. Bacteria contamination of the root canal system must be prevented during and after endodontic treatment.

Once root–canal treatment is completed, immediate restoration of the tooth is recommended whenever possible. If not possible, the root– canal system should be protected by sealing the canals and floor of the pulp chamber with intracoronal barriers [20].

Bonded materials such as glass-ionomer cement or composite resin are preferred. The canal orifices are countersunk with a round bur, and the floor of the chamber is cleaned of excess gutta–percha and sealer. The chamber floor is etched and primed if a resin material is used, or ‘conditioned’ if using glass–ionomer cement or resin–modified glass ionomer. The barrier material is then placed over the floor of the chamber and cured. The intracoronal barrier protects the root–canal system from contamination during the period of temporization and/or while the long–term restorative is performed [4 cit. by 5].

## Basic principles in the restoration of endodontically treated teeth

Coronal restoration subsequent to the root canal treatment needs taking into consideration that reduced tooth structure resulting from caries or trauma (the most reasons for endodontic therapy) and from cavity preparations has a negative influence on the fracture resistance of teeth. Noncarious lesions (abrasion, erosion or abfraction), are also important factors, especially related to the patient's age. The presence of extensive access opening preparations and endodontic therapy itself are the primary reasons for tooth fragility, resulting in partial or complete fractures of tooth cusps or incisal margins and even root fractures [21].

Teeth can be further weakened not only by endodontic treatment, but by pre-existent restorative procedures that also reduce their strength, as well as by the removal of marginal ridges. Endodontic access associated with removal of pulp chamber walls and root dentin appears to be directly responsible for the greater brittleness of endodontically treated teeth. Other factors that may also influence the fracture resistance of teeth are the alterations in the physical and mechanical properties of dentin, tooth anatomy and its dental arch position [22].

- Cuspal coverage restorations appear to grant higher longevity to posterior teeth with root canal treatment; according to some recent studies, bonded restorations thought to preclude the need for cuspal coverage in such teeth, might provide a short-term strengthening [10,12].
- Bonded restorations represent the main choice for conservatively restoring anterior teeth with minimal loss of tooth structure [ 2].
- Maximum preservation of coronal and radicular sound tooth structure is reccomended; coronal tooth structure should be preserved to provide resistance and retention form, either for conservative bonded restoration, or for the core, which will support the crown. [9,23,24]. The purpose of a core is to provide the compromised crown of the tooth with resistance, retention, and geometric form for the final restoration. The core material fills the pulp chamber and replaces lost tooth structure prior to crown preparation. The amount of remaining radicular tooth structure is important for the choice and placement of a post.
- When a tooth has more than 50% of its coronal structure missing, the use of a post–and–core foundation is recommended prior to prosthetic restoration. The main purpose of a post is to retain a core buildup in a tooth with extensive loss of coronal tooth structure.
- Since posts do not reinforce endodontically treated teeth, they are indicated only when there is inadequate tooth structure to retain a core; however, preparation of a post space adds a certain degree of risk to a restorative procedure.
- Posts can either be prefabricated or custom made. Custom cast posts and cores allow for a close adaptation of posts to the post space preparations and should fit optimally [25]. Prefabricated posts have an advantage in that the post space can be prepared and the post directly bonded in a single appointment.
- A ferrule, defined as ‘a metal band or ring used to fit around the root or crown of a tooth’, [13] is highly desirable when a post is used. An adequate ferrule is considered to need a minimum of 2 mm of vertical height and 1 mm of dentin thickness.

**Figure 1:**
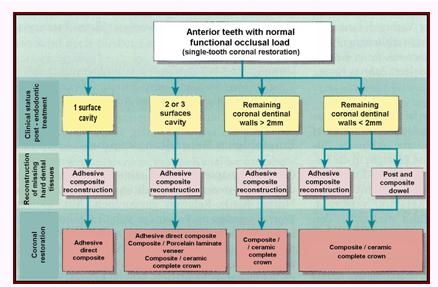
Coronal restoration possibilities for anterior ETT with normal functional occlusal load – Adapted and modified from: Weigl P, Heidemann D. Restaurative Therapie der endodotisch behandelten Zahnes. In: Heidemann D. (Hrsg.): Praxis der Zahnheilkunde. Endodontie. Urban and Fischer Bei Elsevier, Munchen,2001; 24–276.

**Figure 2:**
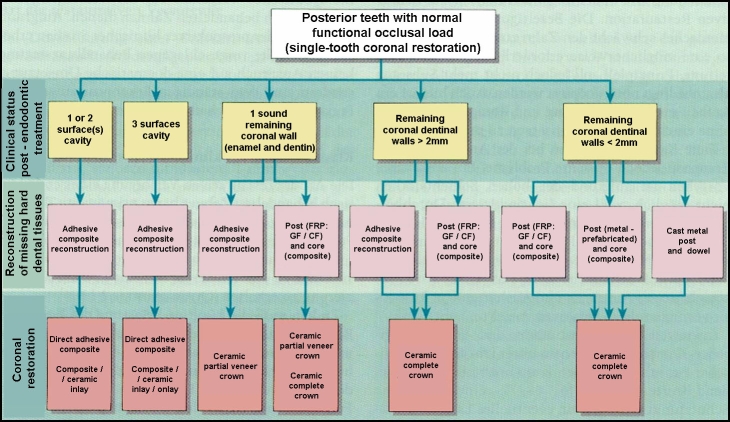
Coronal restoration possibilities for posterior ETT with normal functional occlusal load – Adapted and modified from: Weigl P, Heidemann D. Restaurative Therapie der endodotisch behandelten Zahnes. In: Heidemann D. (Hrsg.): Praxis der Zahnheilkunde. Endodontie. Urban and Fischer Bei Elsevier, Munchen,2001; 24–276

**Figure 3:**
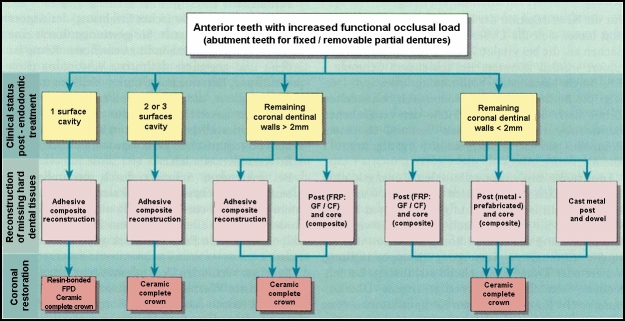
Coronal restoration possibilities for anterior ETT with increased functional occlusal load – Adapted and modified from: Weigl P, Heidemann D. Restaurative Therapie der endodotisch behandelten Zahnes. In: Heidemann D. (Hrsg.): Praxis der Zahnheilkunde. Endodontie. Urban and Fischer Bei Elsevier, Munchen,2001; 24–276

**Figure 4:**
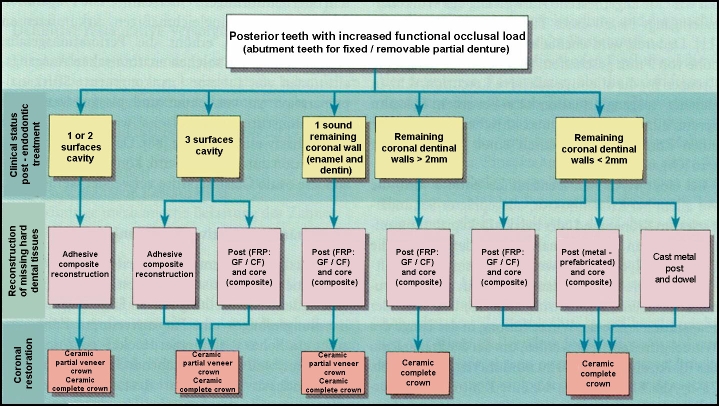
Coronal restoration possibilities for posterior ETT with increased functional occlusal load – Adapted and modified from: Weigl P, Heidemann D. Restaurative Therapie der endodotisch behandelten Zahnes. In: Heidemann D. (Hrsg.): Praxis der Zahnheilkunde. Endodontie. Urban and Fischer Bei Elsevier,Munchen,2001; 24–276

According to these principles, an overview of the coronal restoration possibilities for the endodontically treated teeth (ETT), within the oral rehabilitation treatment plan is presented in Figure 1,Figure 2, Figure 3,Figure 4 related to their clinical status: single-tooth reconstruction or abutment teeth [26].

## Conclusions

Until an endodontically treated tooth is restored to full function, treatment is incomplete. The unrestored endodontically treated tooth is susceptible to fracture, which could lead to loss of the tooth.

Maximum preservation of healthy tooth structure and use of restorative materials with mechanical properties similar to dental structure favor greater longevity of the tooth–restoration complex. In this context, endodontically treated teeth are considered more susceptible to fracture than sound teeth, primarily because of internal tooth structure removal during endodontic therapy [22].

Endodontically treated teeth may provide effective occlusal contact during chewing. They are able to return to a level of masticatory function that is similar to that in natural teeth.

Literature data confirm that coronal leakage is a significant etiology in endodontic failure. Consequently to saliva exposure, leakage will compromise the gutta–percha seal, and the tooth may require retreatment. Because modern endodontic therapy achieves a predictably high success rate, postponing restoration for extended periods of time to be certain of endodontic success is unnecessary and could place the tooth at risk.

There are few data in the literature analyzing the reasons for extraction of endodontically treated teeth. The most common reason found (44%) was a restorative consideration [27]. The survival or functionality of the endodontically treated tooth is currently the emerging aspect of endodontic treatment outcome, rather than healing [28].
